# Mitochondrial genomic variation and phylogenetic relationships of three groups in the genus *Scaphoideus* (Hemiptera: Cicadellidae: Deltocephalinae)

**DOI:** 10.1038/s41598-017-17145-z

**Published:** 2017-12-04

**Authors:** Yimin Du, Wu Dai, Christopher H. Dietrich

**Affiliations:** 1 Key Laboratory of Plant Protection Resources and Pest Management of the Ministry of Education, College of Plant Protection, Northwest A&F University, Yangling, 712100 Shaanxi China; 2 Illinois Natural History Survey, Prairie Research Institute, University of Illinois, 1816 S Oak St., Champaign, IL 61820 USA

## Abstract

The widespread leafhopper genus *Scaphoideus* Uhler is the most diverse genus in Scaphoideini and includes some species that are serious pests and vectors of plant pathogens. Here the first *Scaphoideus* mitogenome sequences are provided for three species, *S. maai*, *S. nigrivalveus* and *S. varius*, representing three main species groups in the Oriental region based on color pattern. The lengths of these three mitogenomes were 15,188, 15,235 and 15,207 bp, respectively. Gene order of three mitogenomes is highly conserved and identical to that of the putative ancestral insect. All three mitogenomes exhibited similar AT nucleotide bias, AT-, GC-skews and codon usage. One large 101 bp intergenic spacer between *trnY* and *cox1* was in *S. varius*. All 22 tRNA genes had typical cloverleaf secondary structures, except for *trnS1* (AGN) which appears to lack the dihydrouridine arm. Genes *atp8*, *nad6* and *nad2* were highly variable while *cox1* showed the lowest nucleotide diversity. Phylogenetic analyses of three concatenated nucleotide datasets using maximum likelihood and Bayesian methods, comprising all 13 mitogenomes currently available for Membracoidea plus mitogenomes for eight outgroup species representing other cicadomorphan superfamilies, yielded the same topology in which *Scaphoideus* species formed a monophyletic group within a larger clade comprising three other included Deltocephalinae.

## Introduction

Insect mitochondrial genomes are typically small double-stranded circular molecules containing 37 genes including 13 protein-coding genes (PCGs), two ribosomal RNAs (rRNAs), 22 transfer RNAs (tRNAs) genes, and one non-coding A + T-rich region (or control region, CR)^1,2^. Owing to its high genome copy numbers, multiple genome-level characteristics, relatively high evolutionary rate and greater phylogenetic informativeness than single mitochondrial genes, mitogenome sequences have been widely used in the comparative and evolutionary genomics, molecular evolution, phylogenetics and population genetics although such data remain sparse or unavailable for many insect groups^2–6^.

One such group, Deltocephalinae, the largest subfamily of leafhoppers, is distributed worldwide and contains the majority of leafhopper vectors of economically important plant diseases. The tribe Scaphoideini, comprising over 630 species in 61 genera, is one of the more diverse, widespread and economically important groups within the subfamily Deltocephalinae. Although the circumscription and morphological characterization of Scaphoideini Oman were substantially revised by Zahniser & Dietrich^7^, the phylogenetic relationships among genera and species of this tribe have not been explored.

The type genus *Scaphoideus* Uhler is the largest and most diverse genus in this tribe, comprising over 200 described species in all major biogeographic regions and differing widely in color and male genitalia. Although some authors attempted to define the genus, its classification remains unsatisfactory. Ball divided the species of the genus into three groups based on face color^8^. DeLong divided it into three distinct subgenera according to male genitalia^9^. However, these two classifications applied only to North American species and have not been widely adopted. Recently, the Oriental species of the genus were also divided into three groups according to coloration^10^. Until now, phylogenetic studies, utilizing morphological and molecular data, have focused more broadly on Deltocephalinae and included few representatives of Scaphoideini^7,11,12^. The small amounts of DNA sequence data currently available for this tribe in GenBank mainly include DNA barcodes of partial *cox1* sequences. No species of Scaphoideini has had its mitochondrial genome sequenced so far. To further elucidate the phylogenetic status and relationships of Scaphoideini, much more data are needed.

Here, we analyze the first three complete mitogenomes for Scaphodeini, based on three Oriental species of the genus *Scaphoideus* representative of the three main color forms found among the Asian fauna: *S. maai*, a species representing the common group with a median longitudinal yellowish or whitish stripe; *S. nigrivalveus*, representing the group with transverse bands on the head, pronotum and scutellum; and *S. varius*, representing the group with dark brown spots or bands on vertex and longitudinal bands on pronotum and scutellum. General genome features including base composition and codon usage of PCGs were compared to explore the sequence variability among these three groups, and tRNA secondary structures were predicated. To examine the phylogenetic utility of complete mtDNA sequences, we reconstructed the phylogenetic relationships among the three newly sequenced species and other leafhoppers for which mtDNA genome data are available, using the concatenated nucleotide sequences of 13 protein-coding genes (PCGs) and two ribosomal RNA genes.

## Results

### General features of three *Scaphoideus* mitogenomes

**Figure 1:**
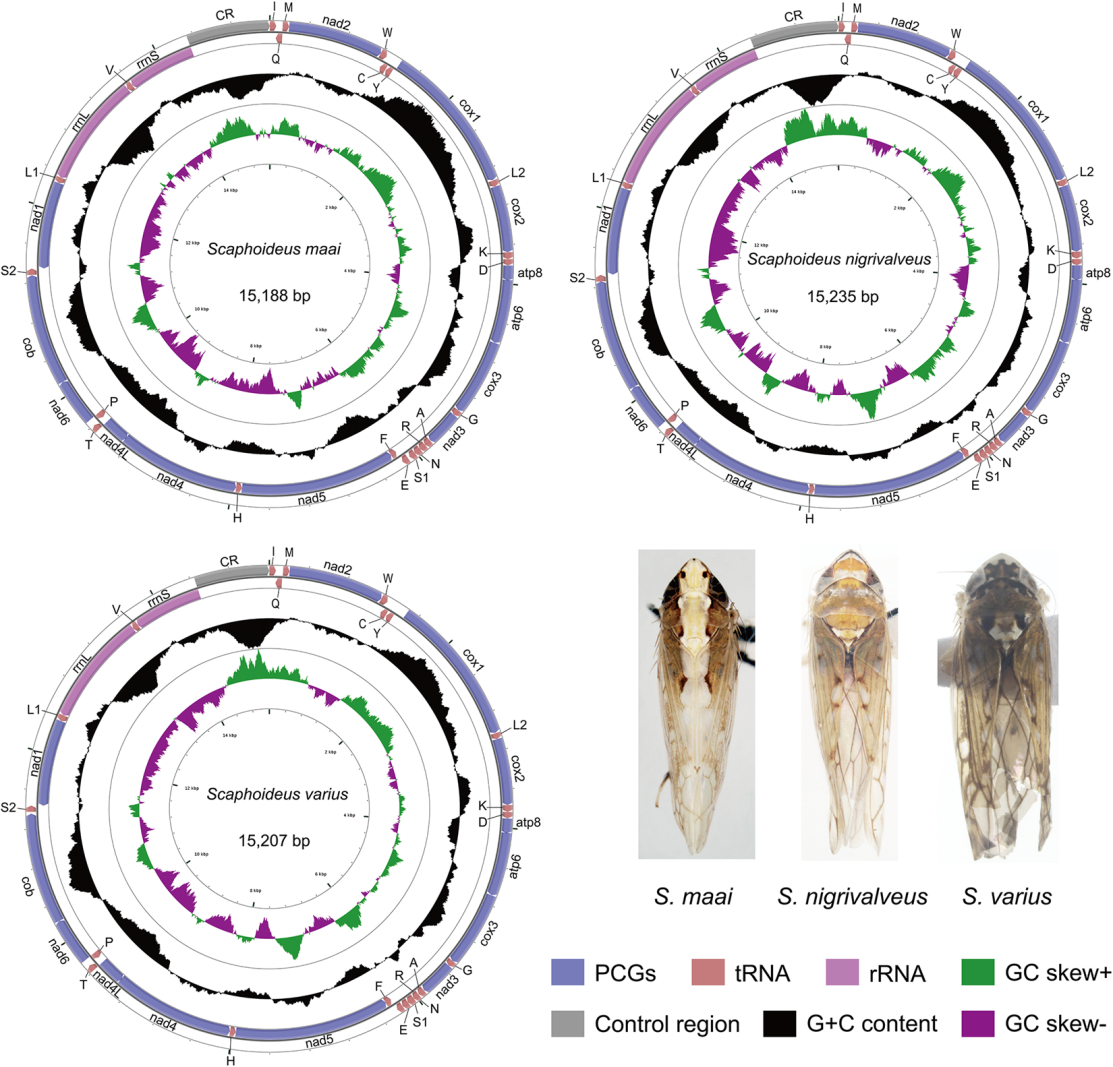
Mitochondrial genomes of three sequenced *Scaphoideus* leafhoppers. Protein coding and ribosomal genes are shown with standard abbreviations. Transfer RNA (tRNA) genes are indicated using the IUPAC-IUB single letter amino acid codes (L1:CUN; L2:UUR; S1:AGN; S2:UCN).

The sizes of the three *Scaphoideus* mitogenomes were 15,188 bp in *S. maai* (GenBank: KY817243), 15,235 bp in *S. nigrivalveus* (GenBank: KY817244) and 15,207 bp in *S. varius* (GenBank: KY817245), respectively. Circular maps of three mitogenomes are shown in the Fig. 1. Each newly sequenced mitogenome contained a typical set of 37 mitochondrial genes (13 PCGs, 22 tRNAs and two rRNAs) and one control region (Supplementary Tables S1–S3). Gene order was invariant and identical to that of *Drosophila yakuba* and to most other previously sequenced Membracoidea^1,2,13–15^.

### Base composition

All three *Scaphoideus* mitogenomes exhibited heavy AT nucleotide bias, with 77.2%, 76.5% and 75.9% in *S. maai*, *S. nigrivalveus* and *S. varius* respectively. The A + T content of the CR (mean value = 82.8%) was always significantly higher (*P* = 0.000) than in other regions, while PCGs showed the lowest A + T content values (mean value = 75.6%) (Supplementary Fig. S1). All three species had higher A + T content in *rrnL* than *rrnS*, with significantly different mean values (80.2% and 77.9%) (Table 1; Supplementary Fig. S1). All three mitogenomes showed positive AT-skews (0.075 to 0.093) and negative GC-skews (−0.154 to −0.178). CR sequences always were slightly T-skewed (−0.014 to −0.026) and C-skewed (−0.006 to −0.100), while the two rRNA genes were moderately T-skewed (−0.102 to −0.130) and markedly G-skewed (0.178 to 0.300) (Table 1).

**Table 1:** Nucleotide compositions, AT- and GC-skew in different regions of three sequenced *Scaphoideus* mitochondrial genomes.

Species	whole		PCGs		*rrnL*		*rrnS*		tRNA		CR	
	length	AT%	length	AT%	length	AT%	length	AT%	length	AT%	length	AT%
*S. maai*	15188	77.2	10929	76.3	1199	81.2	739	78.6	1435	76.8	847	81.9
*S. nigrivalveus*	15235	76.5	10934	75.7	1204	79.8	743	77.7	1440	76.1	902	83.1
*S. varius*	15207	75.9	10938	74.7	1203	79.7	743	77.4	1439	76.1	762	83.4
	AT-skew	GC-skew	AT-skew	GC-skew	AT-skew	GC-skew	AT-skew	GC-skew	AT-skew	GC-skew	AT-skew	GC-skew
*S. maai*	0.083	−0.158	0.090	−0.161	−0.121	0.181	−0.102	0.178	0.049	−0.112	−0.026	−0.100
*S. nigrivalveus*	0.093	−0.154	0.099	−0.16	−0.120	0.228	−0.130	0.193	0.067	−0.071	−0.023	0.018
*S. varius*	0.075	−0.178	0.079	−0.178	−0.127	0.300	−0.106	0.274	0.043	−0.054	−0.014	−0.006

### Protein-coding genes and codon usage

Of the 13 PCGs, nine were located on the majority strand (J-strand) while the other four PCGs were encoded by the minority strand (N-strand). The third codon position had a significantly higher (*P* = 0.000) A + T content than that of the first and second positions (87.5% versus 71.2% and 67.9%) (Supplementary Fig. S1). Pairwise comparisons among the three *Scaphoideus* species (Table 2) indicate that, except for *cox2*, PCGs had fewer variable sites between *S. maai* and *S. nigrivalveus* than species pairs.

**Table 2:** Analyses of polymorphic sites among *S. maai* (*SM*), *S. nigrivalveus* (*SN*) and *S. varius* (*SV*).

PCGs	Variable sites (bp)			Synonymous changes (bp)			Replacement changes (bp)		
	*SM*-*SN*	*SM*-*SV*	*SN*-*SV*	*SM*-*SN*	*SM*-*SV*	*SN*-*SV*	*SM*-*SN*	*SM*-*SV*	*SN*-*SV*
*atp6*	116	140	137	70	78	88	46	62	49
*atp8*	33	35	43	14	16	15	19	19	28
*cob*	176	225	194	130	166	151	46	59	43
*cox1*	205	256	213	177	219	190	28	37	23
*cox2*	128	124	98	84	80	71	44	44	27
*cox3*	154	164	151	100	106	107	54	58	44
*nad1*	138	172	165	97	113	110	41	59	55
*nad2*	182	240	244	88	120	122	94	120	122
*nad3*	68	75	73	37	34	41	31	41	32
*nad4*	222	297	267	131	158	160	91	139	107
*nad4l*	38	45	49	24	22	30	14	23	19
*nad5*	317	353	363	182	200	222	135	153	141
*nad6*	94	129	113	45	62	55	49	67	58

Except for *nad5*, which began with TTG, all other PCGs started with the standard ATN codons, as in other leafhopper mitogenomes (*Tambocerus* sp., *Idioscopus nitidulus*) (Table 3). Most of the PCGs terminated with a TAA stop codon, while *atp6* in *S. maai*, *cox1* and *cob* in *S. nigrivalveus* end with a TAG codon. Two PCGs (*cox2* and *nad4*) terminated with truncated T stop codons in all three species; *nad5* in *S. maai* and *nad1* in *S. varius* also end with the incomplete codon T (Table 3).

**Table 3:** Comparison of length, start and stop codons of 13 protein coding genes (PCGs) among three *Scaphoideus* mitogenomes.

PCGs	*S*. *maai*			*S*. *nigrivalveus*			*S*. *varius*		
	start codon	stop codon	length (bp)	start codon	stop codon	length (bp)	start codon	stop codon	length (bp)
*nad2*	ATA	TAA	975	ATA	TAA	975	ATA	TAA	978
*cox1*	ATG	TAA	1,536	ATG	TAG	1,536	ATG	TAA	1,536
*cox2*	ATA	T-	682	ATA	T-	682	ATA	T-	682
*atp8*	ATT	TAA	153	ATT	TAA	153	ATA	TAA	153
*atp6*	ATG	TAG	654	ATG	TAA	654	ATG	TAA	654
*cox3*	ATG	TAA	780	ATG	TAA	780	ATG	TAA	780
*nad3*	ATT	TAA	354	ATC	TAA	354	ATT	TAA	354
*nad5*	TTG	T-	1,666	TTG	TAA	1,668	TTG	TAA	1,668
*nad4*	ATG	T-	1,309	ATG	T-	1,309	ATG	T-	1,309
*nad4l*	ATT	TAA	273	ATT	TAA	276	ATT	TAA	276
*nad6*	ATA	TAA	477	ATT	TAA	477	ATA	TAA	480
*cob*	ATG	TAA	1,137	ATG	TAG	1,137	ATG	TAA	1,137
*nad1*	ATA	TAA	933	ATA	TAA	933	ATT	T-	931

**Figure 2:**
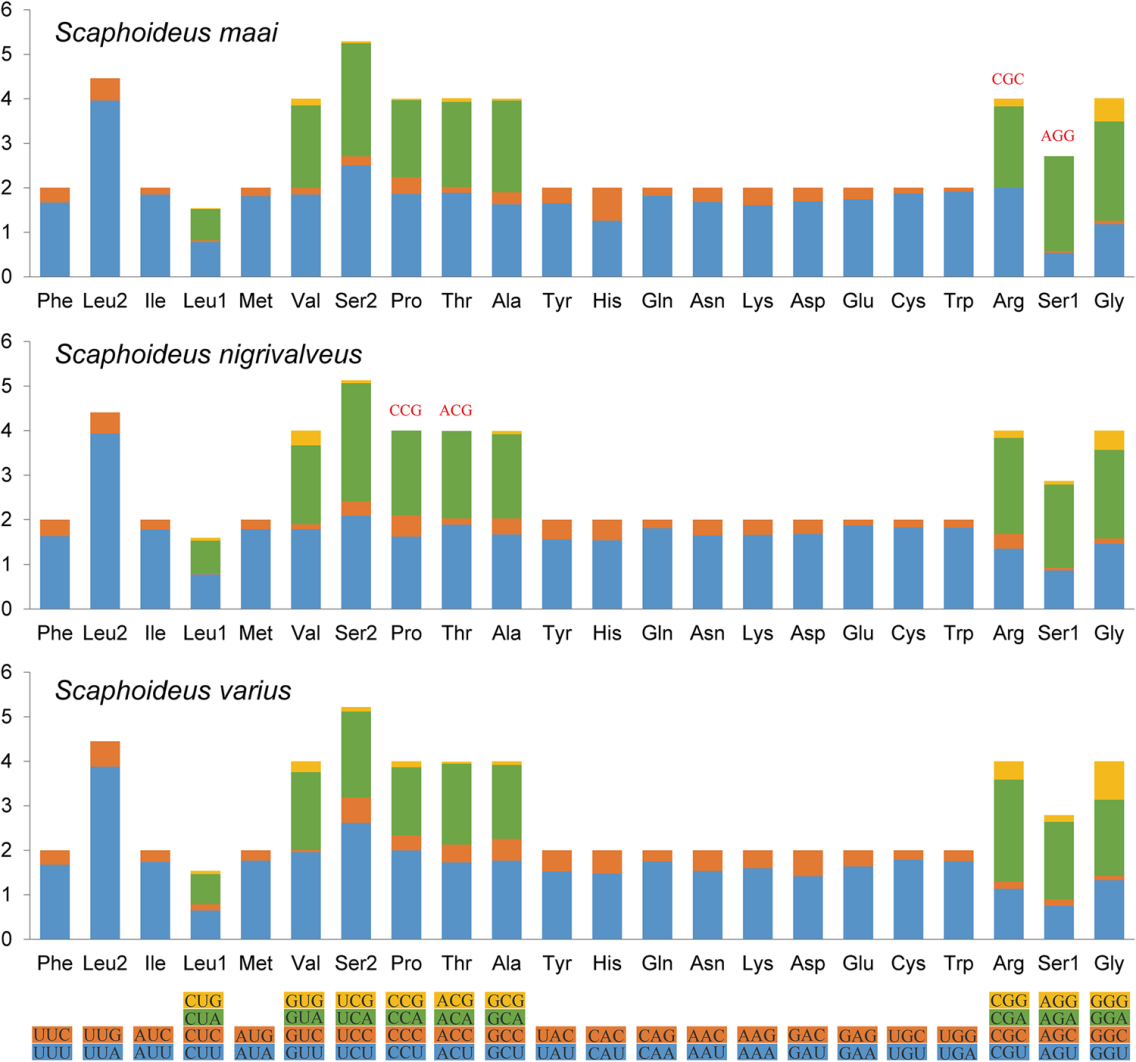
Relative synonymous codon usage (RSCU) of three sequenced *Scaphoideus* mitochondrial genomes. The stop codon is not given. Codons absent in mitogenomes are shown at the top of columns.

After excluding the stop codons, the relative synonymous codon usage (RSCU) was calculated and summarized in Fig. 2. The total numbers of non-stop codons were 3632, 3633 and 3635 in *S. maai*, *S. nigrivalveus* and *S. varius* respectively. Leucine (Leu), Serine (Ser), Isoleucine (Ile) and Methionine (Met) were the most frequently used amino acids. Within each amino acid codon, third positions ending with A/T were more frequent than those terminated with G/C, causing the highest A + T content to occur in third positions. The codons Arg (CGC) and Ser1 (AGG) were missing in *S. maai*, and Pro (CCG) and Thr (ACG) were missing in *S. nigrivalveus*, while *S. varius* had the full 62 available codons (Fig. 2).

### Transfer RNA and ribosomal RNA genes

For the 22 typical animal tRNA genes in each *Scaphoideus* mitogenome, 14 tRNAs were encoded by the J-strand and the remaining eight were located on the N-strand, ranging from 61 to 71 bp in length. *TrnM*, *trnK* and *trnI* showed the highest identical sites percentage when aligned using MAFFT (92.5%, 90.1% and 89.6%, respectively), while *trnY* presented the lowest similarity (62.5%) (Table 4). All tRNAs could be folded into the canonical cloverleaf secondary structure except for *trnS1* (AGN), which lacks the dihydrouridine (DHU) arm and instead forms a loop in all three *Scaphoideus* leafhoppers (Figs S2–S4). Besides the classic A-U and C-G pairs in the secondary structure, there were 27, 25 and 19 G-U base pairings in *S. maai*, *S. nigrivalveus* and *S. varius* respectively. Some other mismatched base pairs (A-A, U-U, A-G and A-C) were also found in the acceptor arm and anticodon arm (Figs S2–S4).

**Table 4:** Identical Sites and its percentage of each tRNA gene alignments.

tRNA	*trnA*	*trnC*	*trnD*	*trnE*	*trnF*	*trnG*	*trnH*	*trnI*	*trnK*	*trnL1*(CUN)	*trnL2*(UUR)
Identical Sites	47	54	46	47	44	52	50	60	64	52	50
Percentage (%)	70.1	84.4	69.7	71.2	63.8	80	79.4	89.6	90.1	77.6	75.8
tRNA	*trnM*	*trnN*	*trnP*	*trnQ*	*trnR*	*trnT*	*trnV*	*trnW*	*trnY*	*trnS1*(AGN)	*trnS2*(UCN)
Identical Sites	62	56	57	53	48	55	50	53	45	52	52
Percentage (%)	92.5	77.8	81.4	76.8	70.6	85.9	76.9	74.6	62.5	77.6	77.6

The lengths of the two rRNA genes (*rrnL* and *rrnS*) in the *Scaphoideus* mitogenomes were about 1200 and 740 bp, with the mean A + T contents of 80.2% and 77.9% respectively (Table 1), and both *rrn* genes were encoded on the N-strand. The large rRNA subunit was located at a conserved position between *trnL1* (CUN) and *trnV*, while the small rRNA subunits was between *trnV* and the control region (Fig. 1). The percentage of pairwise identity between the three *Scaphoideus* species based on the MAFFT alignment is summarized in Table 5. As for the PCGs *S. maai* was more similar to *S. nigrivalveus* than to *S. varius* in both *rrnL* or *rrnS* (80.4% versus 78.3%, 84.3 versus 79.4%, respectively) (Table 5).

**Table 5:** Pairwise identity (%) of two rRNA genes and control region among *S. maai* (*SM*), *S. nigrivalveus* (*SN*) and *S. varius* (*SV*).

	*SM-SN-SV*	*SM-SN*	*SM-SV*	*SN-SV*
*rrnL*	77.7%	80.4%	78.3%	76.9%
*rrnS*	81.4%	84.3%	79.4%	81.1%
Control region	48.2%	54.4%	51.7%	50.5%

### Gene overlaps and non-coding regions

All three *Scaphoideus* mitogenomes had gene overlaps and each single overlap ranged from 1 to 8 bp. *S. maai* had a total of 31 bp in overlaps between 10 gene junctions, while *S. nigrivalveus* and *S. varius* had 34 bp overlaps between 11 gene junctions and 30 bp overlaps between nine gene junctions, respectively (Supplementary Tables S1–S3). Except for the two existing common pairs of gene overlaps: *atp8*-*atp6* (7 bp) and *nad4*-*nad4l* (7 bp) which are found in other leafhoppers (*D. nuchalis*, *E. vitis*, *H. vitripennis*, *I. nitidulus*, *N. cincticeps*), all three *Scaphoideus* species also share the same five other pairs of gene overlaps: *trnR*-*trnN* (1 bp), *trnN*-*trnS1* (AGN) (1 bp), *nad6*-*cob* (1 bp), *trnI*-*trnQ* (3 bp) and *trnW*-*trnC* (8 bp) (Supplementary Tables S1–S3).

Excluding the control region, there were 10, nine and 13 intergenic spacers totaling 70, 46 and 152 bp non-coding bases in *S. maai*, *S. nigrivalveus* and *S. varius* respectively. All three mitogenomes have the same two intergenic spacer patterns, between *nad4l*-*trnT* (2 bp) and *trnP*-*nad6* (2 bp) respectively. The longest intergenic spacers in *Scaphoideus* mitogenomes were present between *trnY* and *cox1* (with 26, 21 and 101 bp respectively). A much larger, 101 bp, intergenic spacer in *S. varius* made it distinctly different from other two species (Supplementary Tables S1–S3). We confirmed the presence of this spacer by Sanger sequencing using primers (Forward 5′-3′: CGTTTAGCTTTTACTTC and Reverse 5′-3′: GTTCCAGGGTGTGCTAAT) located in flanking regions of *nad2* and *cox1*.

The putative control region, or A + T rich region, located between *rrnS* and *trnI*, was the most variable region in the whole mitogenome, with the pairwise identity between *Scaphoideus* species were relatively low (all < 55%) (Table 5). The full lengths of CR in three *Scaphoideus* mitogenomes were 847, 902 and 762 bp respectively, comparable to other sequenced leafhoppers (from 399 bp in *N. cincticeps* to 1581 bp in *Tambocerus* sp.). No tandem repeat units were found in *Scaphoideus* species.

### Nucleotide diversity among three *Scaphoideus* mitogenomes

**Figure 3:**
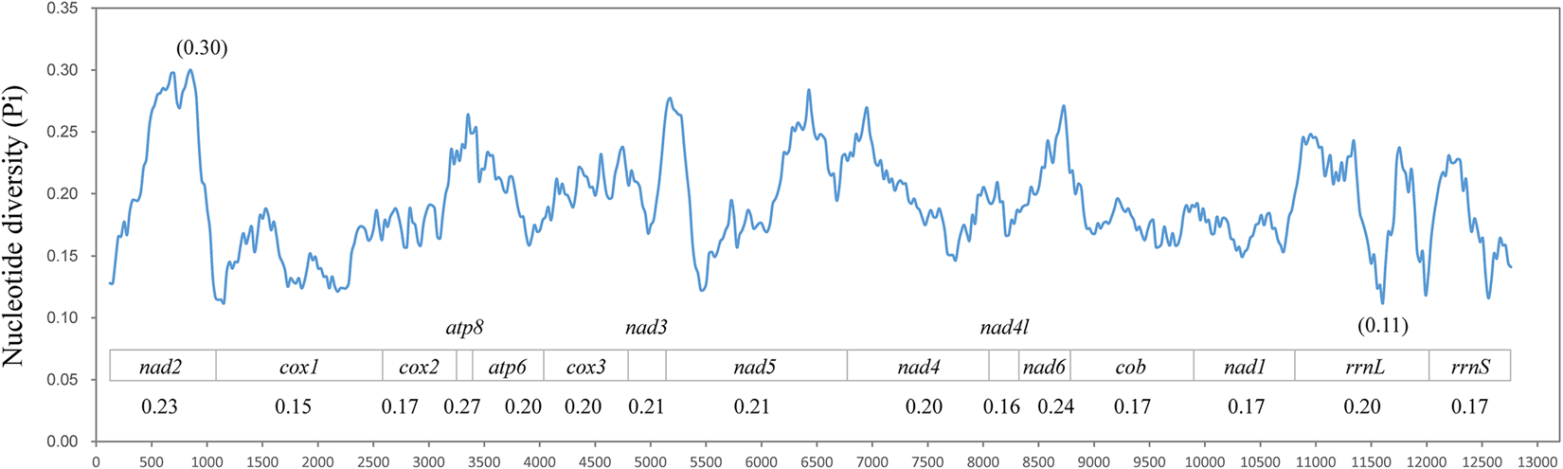
Sliding window analyses of protein coding genes and ribosomal RNA genes among three *Scaphoideus* mitogenomes. The blue line was drawn using each mid-point value with its nucleotide diversity (Pi) (a sliding window of 250 bp with the step size of 25 bp). Pi value of each PCG was shown under the gene name.

Nucleotide diversity of the 13 PCGs and two rRNA genes among three *Scaphoideus* mitogenomes were shown in Fig. 3. The most variable region (729–978) was in *nad2* (Pi = 0.30) while the most conserved fragment (11475–11730) was in *rrnL* (Pi = 0.11). Within each gene, the shortest PCG *atp8* (153 bp, Pi = 0.27) present the highest variability, *nad6* (Pi = 0.24) and *nad2* (Pi = 0.23) were also highly variable. The most conserved PCGs were *cox1* (Pi = 0.15) and *nad4l* (Pi = 0.16). Two rRNA genes were highly conserved, with the value 0.20 in *rrnL* and 0.17 in *rrnS* respectively.

### Phylogenetic relationships

**Figure 4:**
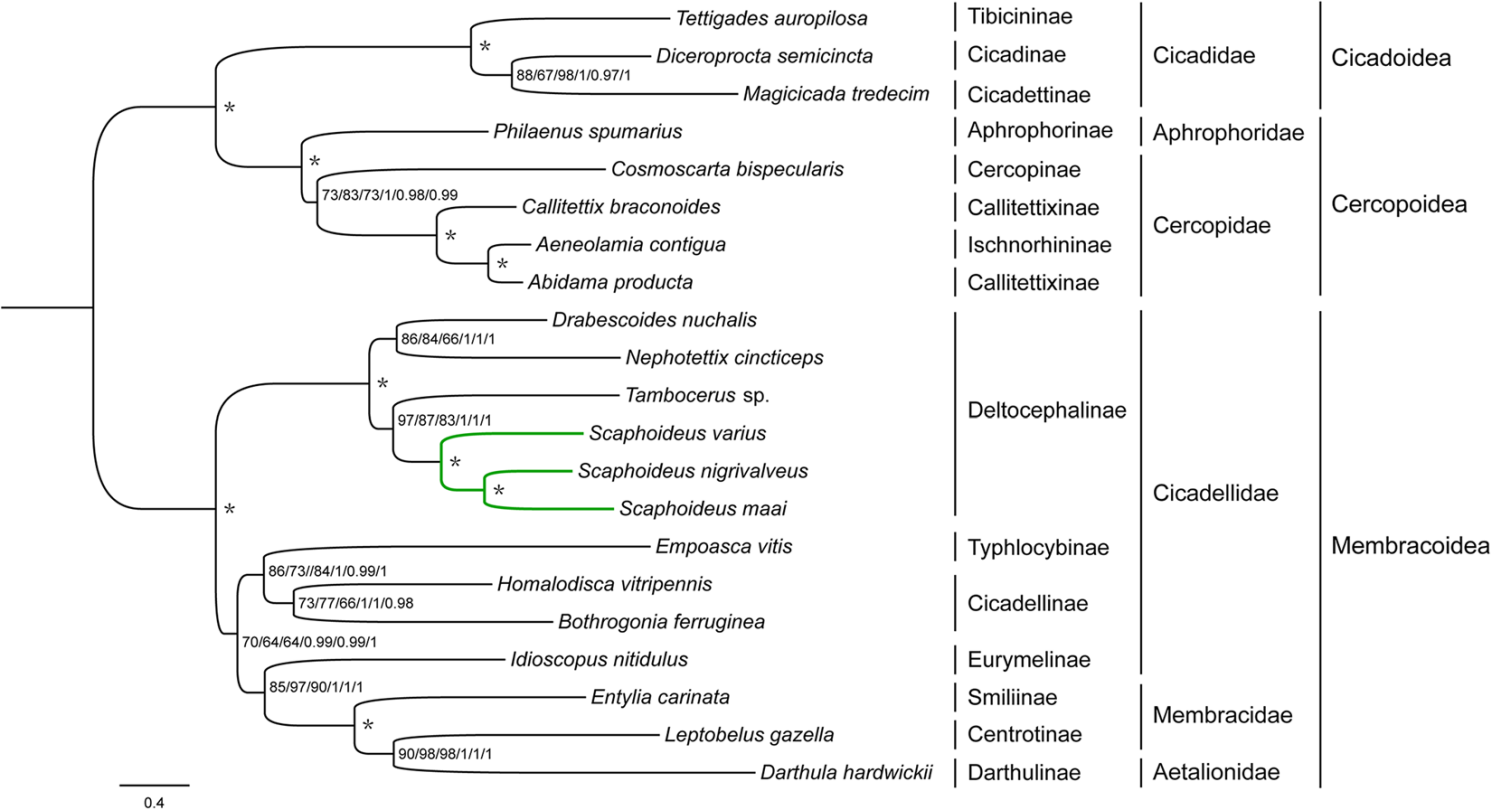
Phylogenetic relationships for *Scaphoideus* based on the P123/P123DEGEN/P123R datasets inferred from RaxML and MrBayes. Numbers on branches are Bootstrap values (BS) and Bayesian posterior probabilities (PP) (ML-P123; ML-P123DEGEN; ML-P123R; BI-P123; BI-P123DEGEN; BI-P123R, respectively). An asterisk indicates BS = 100 and PP = 1.0 in all six inferences.

In this study, no saturation was detected for the three candidate nucleotide sequence datasets (P123, P123DEGEN and P123R) prepared for ML and BI analyses (all *Iss* < *Iss.cSym* or *Iss.cAsym*, *P < *0.05) (Table 6), suggesting that the concatenated data were suitable for phylogenetic analysis. Thus, we analyzed these three different datasets, in addition to the corresponding amino acid sequence data, to evaluate the tree topology and nodal support. The phylogenetic tree topologies yielded by all six analyses of nucleotide sequence data were identical to each other (Fig. 4). A separate analysis of amino acid sequence data yielded a topology that was identical except for two deep internal nodes within Membracoidea that received only low to moderate ML bootstrap support in all analyses (Fig. S5). The lower overall branch support for the tree resulting from analysis of amino acid sequences indicates that the nucleotide sequence data contain phylogenetic signal (e.g., in third codon positions) that is lost when the data are translated to amino acids.

**Table 6:** Substitution saturation tests for each dataset.

Dataset	Observed *Iss*	*Iss.cSym* ^a^	*Psym* ^b^	*Iss.cAsym* ^c^	*Pasym* ^d^
P123	0.4236	0.8448	0.0000	0.6447	0.0000
P123DEGEN	0.5079	0.8448	0.0000	0.6447	0.0000
P123R	0.4694	0.8515	0.0000	0.6444	0.0000

^a^critical values assuming a symmetrical tree. ^b^Signifcant difference between *Iss* and *Iss.cSym* (two-tailed test). ^c^critical values assuming an extreme asymmetrical tree. ^d^Signifcant difference between *Iss* and *Iss.cAsym* (two-tailed test).

Monophyly of the three cicadomorphan superfamilies Cicadoidea, Cercopoidea and Membracoidea was strongly supported in all analyses (Bootstrap support values (BS) = 100, Bayesian posterior Probability (PP) = 1.00). All nodes received high support (PP > 0.96) in Bayesian analyses of nucleotide sequence data but a few nodes received only moderate or low support ML analyses of some datasets (BS < 75). Within Cicadellidae, the six Deltocephalinae species constituted one clade and formed a sister group to other leafhoppers. Within Deltocephalinae, *Scaphoideus *species (tribe Scaphoideini) formed a monophyletic sister group to *Tambocerus* sp. (tribe Athysanini) and *Drabescus nuchalis* (tribe Drabescini) was sister to *Nephotettix cincticeps* (tribe Chiasmini). Of the three *Scaphoideus* species, *S. maai* was sister clade to *S. nigrivalveus*, consistent with the relatively high pairwise sequence similarity of these two species.

## Discussion

Consistent with previous observations of Membracoidea, mitogenome sequences of *Scaphoideus *were highly conserved in gene content, gene size, gene order, base composition, codon usage of PCGs and tRNA secondary structures. Variation in the length of mt genomes is mostly due to length variation in the control region, which ranges from 70 bp (Orthoptera: *Ruspoliadubia*)^16^ to 4.6 kb (Diptera: *Drosophila melanogaster*) in insects^17^. The sizes of the A + T-rich region in *S. maai*, *S. nigrivalveus* and *S. varius* are consistent with those of the A + T-rich regions of other leafhoppers, which range from 399 bp in *N. cincticeps* to 1581 bp in *Tambocerus* sp., but are relatively short compared to most other insect mitogenomes. Moreover, the three newly sequenced species share 32.3% sequence identity in this region.

Phylogenetic analyses suggest that the *S. maai* group is more closely related to the *S. nigrivalveus* group than to the *S. varius* group, consistent with the pairwise differences in both PCGs and rRNA genes. While Scaphoideini was found to be relatively close to Drabescini in the phylogeny of Deltocephalinae based on combined morphological, 28 S and Histone H3 data^7^, our analysis placed a species of the poorly defined and polyphyletic tribe Athysanini (*Tambocerus*, not included in Zahniser and Dietrich’s dataset) as sister to *Scaphoideus*. In recent phylogenetic studies of the entire subfamily Deltocephalinae based on partial gene sequences and morphological characters, Scaphoideini was resolved as paraphyletic with respect to Drabescini with low branch support. Further phylogenetic studies are needed to elucidate the status and relationships within this group. Although our taxon sample includes only a small fraction of the diversity of leafhoppers, a group comprising >21,000 known species, our phylogenetic analyses of mitogenome sequences indicate that such data are able to resolve relationships at various levels in the taxonomic hierarchy of this insect group. Thus, addition of taxa to our leafhopper mitogenome dataset may help improve resolution of the still poorly understood relationships among major leafhopper lineages. Interestingly, as in our analysis of mitogenome data, a recent larger-scale phylogenomic study of Membracoidea that included nucleotide sequence data from 388 gene regions was also unable to resolve some deep internal branches of the phylogeny of this group^18^. This suggests that some ancient divergences among leafhoppers may be difficult to resolve, even after the addition of much more data.

Mitochondrial genes have been widely used as genetic markers, especially with *cox1* partial sequences gaining widespread popularity as convenient DNA barcodes for species identification^19^. Our sliding window analysis indicated that *cox1* is one of the most conserved protein-coding genes of the mitogenome and the two rRNA genes are also highly conserved when compared to other regions. Similar results were found in recent studies of Psylloidea and Psychodidae species^20,21^. On the other hand, mitogenome sequence data are sufficiently variable to also have potential use in single nucleotide polymorphism (SNP) studies.

Partial mitogenome sequence data were recently used in a population genetic study of *Scaphoideus*. Papura *et al*. used 10 polymorphic microsatellite loci and a variable 623 bp region of the mitogenome (*trnL2*(UUR) - *cox2*) in an attempt to elucidate the colonization scenario of the phytoplasma vector species *S. titanus*, which is native to northeastern North America but is now well established and spreading in Europe^22^. Consistent with microsatellite data, the mitochondrial data indicated much higher haplotype diversity among the native North American populations than observed in European samples, which displayed low levels of genetic diversity and no isolation by distance^22^. The three most variable coding regions in the mitogenomes of *Scaphoideus* species sequenced for our study were *atp8* (Pi = 0.27), *nad6* (Pi = 0.24) and *nad2* (Pi = 0.23), while *cox2* (Pi = 0.17) was relatively conserved and slightly below the mean Pi value for PCGs (0.20). This suggests that the first three mentioned markers may be more useful than *cox2* for future population genetic studies. Although *atp8* (153 bp) may be too short to be very informative, *nad6* (477 bp) and *nad2* (975 bp) should be considered for use in future *Scaphoideus* population genetic studies.

## Materials and Methods

### Sample collection and DNA extraction

All species used in this study were collected in China between 2011 and 2015 (Table S4). Fresh specimens were captured and preserved in 100% ethanol, and stored at −20 °C in the laboratory. After morphological identification, voucher specimens with male genitalia prepared were deposited in the Entomological Museum of Northwest A&F University, and total genomic DNA was extracted from muscle tissues of the thorax using the DNeasy DNA Extraction kit (Qiagen).

### Next generation sequence assembly

Most of the mitochondrial genome sequences of the three species were generated using Illumina HiSeq™2500 with paired reads of 2 × 150 bp. A total of 23,047,208, 16,162,636 and 26,342,108 raw paired reads were retrieved and quality-trimmed using CLC Genomics Workbench v7.0.4 (CLC Bio, Aarhus, Denmark) with default parameters for *S. maai*, *S. nigrivalveus* and *S. varius* respectively. Subsequently, with the mitochondrial genome of *Drabescus nuchalis* (KR349344)^14^ employed as a bait sequence, the resultant 23,047,153, 16,162,588 and 26,342,065 clean paired reads were used for mitogenome reconstruction using MITObim v1.7 software^23^ with default parameters. A total of 18,361, 15,925 and 18,166 individual mitochondrial reads yielded an average coverage of 120.9 × , 81.9 × and 252.0 × for *S. maai*, *S. nigrivalveus* and *S. varius* respectively.

### Gap closing-PCR amplification and sequencing

According to the flanking sequences assembled from the NGS data, we designed three pairs of primers to amplify the control region (CR) (*S. maai*: Forward 5′-3′: TAGGGTATCTAATCCTAGTTTA and Reverse 5′-3′: TGTTGATGCTACTCTTTG; *S. nigrivalveus*: Forward 5′-3′: CGCCAAATTCTTTGAGCT and Reverse 5′-3′: ATTGTGAAATGGTGCTGA; *S. varius*: Forward 5′-3′: TTAACCGCGAATGCTGGCAC and Reverse 5′-3′: GAATGGAATAAACGACAG). PCR reactions were performed with TaKaRa LA-Taq Kits (TaKaRa Co., Dalian, China) under the following cycling conditions: 5 min at 94 °C, 38 cycles of 30 s at 94 °C, 1 min at 45–50 °C, 2–3 min at 68 °C, and a final elongation step at 68 °C for 10 min. PCR products were eletrophoresed on 1% agarosegels, purified and then sequenced in both directions on an ABI 3730 XL automated sequencer (Applied Biosystems).

### Genome annotation and bioinformatic analyses

All the three *Scaphoideus* mitochondrial genomes were annotated with GENEIOUS R8 (Biomatters Ltd., Auckland, New Zealand). All 13 protein-coding genes and two rRNA genes were identified by comparison with the homologous sequences of other leafhoppers from GenBank. The 22 tRNA genes were determined using both of the tRNAScan-SE server v 1.21^24^ and MITOS WebSever^25^, the clover-leaf secondary structures were also predicted by the MITOS WebSever.

The base composition and relative synonymous codon usage (RSCU) values of each protein coding gene (PCG) were calculated with MEGA 6.06^26^. Strand asymmetry was calculated using the formulas AT skew = [A−T]/[A + T] and GC skew = [G−C]/[G + C]^27^. Polymorphic sites and nucleotide diversity (Pi) of each PCG among three *Scaphoideus* species were calculated with DnaSP 5.0^28^. A sliding window of 250 bp (in 25 bp overlapping steps) was used to estimate Pi among PCGs and rRNA genes across the alignment of three *Scaphoideus* mitogenomes.

### Phylogenetic analyses

#### Taxa selection

A dataset consisting of the three newly sequenced taxa and 10 previously available Membracoidea mitogenomes (3 treehoppers and 10 leafhoppers), plus outgroups consisting of three species of Cicadoidea and five species of Cercopoidea was compiled for phylogenetic analysis (Table 7).

**Table 7:** List of mitochondrial genomes used for the phylogenetic analysisin this study.

Superfamily	Family	Subfamily	Tribe	Species	Accession number	Reference
Cicadoidea	Cicadidae	Tibicininae	Tettigadini	*Tettigades auropilosa*	KM000129	Direct Submission
		Cicadettinae	Taphurini	*Magicicada tredecim*	KM000130	Direct Submission
		Cicadinae	Cryptotympanini	*Diceroprocta semicincta*	KM000131	Direct Submission
Cercopoidea	Aphrophoridae	Aphrophorinae	Philaenini	*Philaenus spumarius*	AY630340	Stewart and Beckenbach^39^
	Cercopidae	Cercopinae	Cosmoscartini	*Cosmoscarta bispecularis*	KP064511	Yang *et al*.^40^
		Ischnorhininae	Tomaspidini	*Aeneolamia contigua*	JX844626	Liu *et al*.^41^
		Callitettixinae	Callitettixini	*Abidama producta*	GQ337955	Liu *et al*.^41^
				*Callitettix braconoides*	JX844628	Liu *et al*.^41^
Membracoidea	Membracidae	Centrotinae	Leptobelini	*Leptobelus gazella*	JF801955	Zhao and Liang, ^13^
		Smiliinae	Polyglyptini	*Entylia carinata*	KX495488	Mao *et al*.^42^
	Aetalionidae	Darthulinae	Darthulini	*Darthula hardwickii*	KP316404	Liang *et al*.^43^
	Cicadellidae	Cicadellinae	Proconiini	*Homalodisca vitripennis*	AY875213	Direct Submission
			Cicadellini	*Bothrogonia ferruginea*	KU167550	Direct Submission
		Typhlocybinae	Empoascini	*Empoasca vitis*	KJ815009	Zhou *et al*.^44^
		Eurymelinae	Idiocerini	*Idioscopus nitidulus*	KR024406	Direct Submission
		Deltocephalinae	Drabescini	*Drabescoides nuchalis*	KR349344	Wu *et al*.^14^
			Chiasmini	*Nephotettix cincticeps*	KP749836	Direct Submission
			Athysanini	*Tambocerus sp*.	KT827824	Yu *et al*.^15^
			Scaphoideini	*Scaphoideus maai*	KY817243	This study
				*Scaphoideus nigrivalveus*	KY817244	This study
				*Scaphoideus varius*	KY817245	This study

#### Sequence alignment and substitution saturation test

Each of the 13 PCGs and two rRNA genes was aligned separately based on the invertebrate mitochondrial genetic code using the MAFFT algorithm in the TranslatorX online server (http://translatorx.co.uk/)^29^, with poorly aligned sites removed from the protein alignment before back-translating to nucleotides using GBlocks under default settings. Each of the two rRNAs was aligned using the Q-INS-i method through MAFFT version 7 alignment server (http://mafft.cbrc.jp/alignment/server/)^30^. Potential substitution saturation of each dataset was assessed using the index of substitution saturation (*Iss*) of Xia *et al*.^31^ implemented in the DAMBE 5^32^.

#### Dataset concatenation, partitioning and substitution model selection

Alignments of individual genes were concatenated using SequenceMatrix 1.7.8^33^. To compensate for nucleotide compositional heterogeneity between taxa, the “Degen” approach was selected to make synonymous changes largely invisible but leaving the inference of non-synonymous change largely intact, as implemented in the online server (http://www.phylotools.com/)^34,35^. To assess the stability of phylogenetic results under strategies for reducing noise in the data, fourdatasets were generated: 1) P123: 13 PCGs with 9912 nucleotides; 2) P123DEGEN: 13 PCGs with all coding positions with 9912 nucleotides; 3) P123R: 13 PCGs and two rRNAs with 12047 nucleotides; 4) AA: amino acid sequences of 13 PCGs with 3304 amino acids. PartitionFinder v1.1.1^36^ was employed to infer the optimal nucleotide substitution models and partition strategies. Data blocks for PCGs were defined by codon positions. The Bayesian information criterion (BIC) was chosen as the metric for the partitioning scheme under the “greedy” search algorithm. Details of the best-fit schemes calculated for each dataset are shown in Supplementary Table S5.

#### Phylogenetic inference

ML analyses were implemented using raxmlGUI 1.5^37^ under the GTRGAMMAI model, and support for nodes was assessed by performing 1000 rapid bootstrap replicates (BS). Bayesian analyses were conducted using MrBayes 3.2.6^38^. Following the partition schemes suggested by PartitionFinder, all model parameters were set as unlinked across partitions. Two simultaneous runs with four independent Markov chains were run for seven million generations and trees were sampled every 1000th generation. After the average standard deviation of split frequencies fell below 0.01, the first 25% of samples were discarded as burn-in and the remaining trees were used to generate a consensus tree and calculate the posterior probabilities (PP).

## Electronic supplementary material

Supplementary file
