# Supplementary material for: Mitochondrial genomic variation and phylogenetic relationships of three groups in the genus Scaphoideus (Hemiptera: Cicadellidae: Deltocephalinae)
Source: Sci Rep. 2017 Dec 4;7:16908. doi: 10.1038/s41598-017-17145-z (PMC5714952; doi:10.1038/s41598-017-17145-z)
Supplement: Supplementary file 1 — Supplementary file [file 41598_2017_17145_MOESM1_ESM.doc]

**Mitochondrial genomic variation and phylogenetic relationships of three groups in the genus *Scaphoideus* (Hemiptera: Cicadellidae: Deltocephalinae)**

Yimin Du^1^, Wu Dai^1*^, Christopher H. Dietrich^2^

^1^ Key Laboratory of Plant Protection Resources and Pest Integrated Management of the Ministry of Education, College of Plant Protection, Northwest A&F University, Yangling, 712100, Shaanxi, China

^2^ Illinois Natural History Survey, Prairie Research Institute, University of Illinois, Champaign, 61820, Illinois, United States of America

*Corresponding Author: daiwu@nwsuaf.edu.cn (WD)


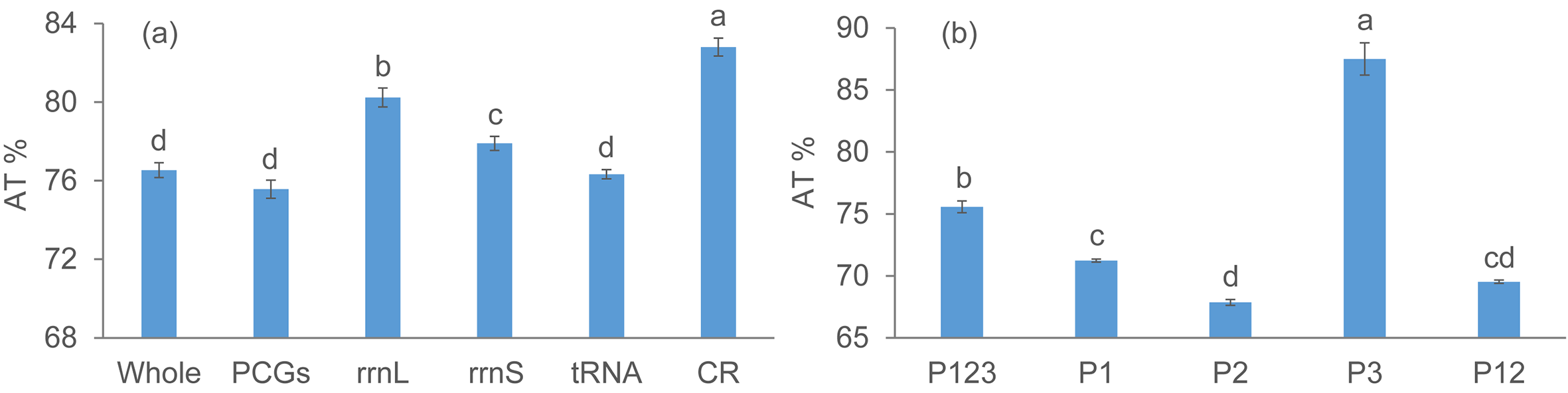


**Figure S1.** (a) Differences in A+T content of different regions of the three sequenced *Scaphoideus* mitogenomes; (b) same for different codon positions. Whole = entire mitogenome; PCGs = protein-coding genes; *rrnL* = large subunit ribosomal RNAs; *rrnS* = small subunit ribosomal RNAs. P123 = protein-coding genes; PCG1 = first codon position; PCG2 = second codon position; PCG3 = third codon position; PCG12 = combined first and second codon positions. (One-way ANOVA analyses, SNK, *P <* 0.05).

**Supplementary Table S1.** Organization of the *Scaphoideus maai* mitochondrial genome.

| Name | Start | Stop | Intergenic nucleotides | Length | Start codon | Stop codon | Anticodon | coding strand |
| --- | --- | --- | --- | --- | --- | --- | --- | --- |
| *trnI* | 1 | 65 | 0 | 65 |  |  | GAT | ＋ |
| *trnQ* | 63 | 131 | -3 | 69 |  |  | TTG | － |
| *trnM* | 131 | 196 | -1 | 66 |  |  | CAT | ＋ |
| *nad2* | 197 | 1,171 | 0 | 975 | ATA | TAA |  | ＋ |
| *trnW* | 1,171 | 1,238 | -1 | 68 |  |  | TCA | ＋ |
| *trnC* | 1,231 | 1,294 | -8 | 64 |  |  | GCA | － |
| *trnY* | 1,301 | 1,364 | 6 | 64 |  |  | GTA | － |
| *cox1* | 1,391 | 2,926 | 26 | 1,536 | ATG | TAA |  | ＋ |
| *trnL2(UUR)* | 2,931 | 2,996 | 4 | 66 |  |  | TAA | ＋ |
| *cox2* | 2,997 | 3,678 | 0 | 682 | ATA | T |  | ＋ |
| *trnK* | 3,679 | 3,748 | 0 | 70 |  |  | CTT | ＋ |
| *trnD* | 3,749 | 3,812 | 0 | 64 |  |  | GTC | ＋ |
| *atp8* | 3,813 | 3,965 | 0 | 153 | ATT | TAA |  | ＋ |
| *atp6* | 3,959 | 4,612 | -7 | 654 | ATG | TAG |  | ＋ |
| *cox3* | 4,614 | 5,393 | 1 | 780 | ATG | TAA |  | ＋ |
| *trnG* | 5,395 | 5,458 | 1 | 64 |  |  | TCC | ＋ |
| *nad3* | 5,459 | 5,812 | 0 | 354 | ATT | TAA |  | ＋ |
| *trnA* | 5,816 | 5,882 | 3 | 67 |  |  | TGC | ＋ |
| *trnR* | 5,883 | 5,949 | 0 | 67 |  |  | TCG | ＋ |
| *trnN* | 5,949 | 6,015 | -1 | 67 |  |  | GTT | ＋ |
| *trnS1(AGN)* | 6,015 | 6,079 | -1 | 65 |  |  | GCT | ＋ |
| *trnE* | 6,102 | 6,166 | 22 | 65 |  |  | TTC | ＋ |
| *trnF* | 6,167 | 6,228 | 0 | 62 |  |  | GAA | － |
| *nad5* | 6,229 | 7,894 | 0 | 1,666 | TTG | T |  | － |
| *trnH* | 7,895 | 7,956 | 0 | 62 |  |  | GTG | － |
| *nad4* | 7,957 | 9,265 | 0 | 1,309 | ATG | T |  | － |
| *nad4l* | 9,259 | 9,531 | -7 | 273 | ATT | TAA |  | － |
| *trnT* | 9,534 | 9,596 | 2 | 63 |  |  | TGT | ＋ |
| *trnP* | 9,597 | 9,663 | 0 | 67 |  |  | TGG | － |
| *nad6* | 9,666 | 10,142 | 2 | 477 | ATA | TAA |  | ＋ |
| *cob* | 10,142 | 11,278 | -1 | 1,137 | ATG | TAA |  | ＋ |
| *trnS2(UCN)* | 11,282 | 11,345 | 3 | 64 |  |  | TGA | ＋ |
| *nad1* | 11,345 | 12,277 | -1 | 933 | ATA | TAA |  | － |
| *trnL1(CUN)* | 12,278 | 12,340 | 0 | 63 |  |  | TAG | － |
| *rrnL* | 12,341 | 13,539 | 0 | 1,199 |  |  |  | － |
| *trnV* | 13,540 | 13,602 | 0 | 63 |  |  | TAC | － |
| *rrnS* | 13,603 | 14,341 | 0 | 739 |  |  |  | － |
| CR | 14,342 | 15,188 | 0 | 847 |  |  |  | ＋ |

**Supplementary Table S2.** Organization of the *Scaphoideus nigrivalveus* mitochondrial genome.

| Name | Start | Stop | Intergenic nucleotides | Length | Start codon | Stop codon | Anticodon | coding strand |
| --- | --- | --- | --- | --- | --- | --- | --- | --- |
| *trnI* | 1 | 65 | 0 | 65 |  |  | GAT | ＋ |
| *trnQ* | 63 | 131 | -3 | 69 |  |  | TTG | － |
| *trnM* | 131 | 197 | -1 | 67 |  |  | CAT | ＋ |
| *nad2* | 198 | 1,172 | 0 | 975 | ATA | TAA |  | ＋ |
| *trnW* | 1,173 | 1,240 | 0 | 68 |  |  | TCA | ＋ |
| *trnC* | 1,233 | 1,294 | -8 | 62 |  |  | GCA | － |
| *trnY* | 1,298 | 1,367 | 3 | 70 |  |  | GTA | － |
| *cox1* | 1,389 | 2,924 | 21 | 1,536 | ATG | TAG |  | ＋ |
| *trnL2(UUR)* | 2,928 | 2,992 | 3 | 65 |  |  | TAA | ＋ |
| *cox2* | 2,993 | 3,674 | 0 | 682 | ATA | T |  | ＋ |
| *trnK* | 3,675 | 3,745 | 0 | 71 |  |  | CTT | ＋ |
| *trnD* | 3,750 | 3,813 | 4 | 64 |  |  | GTC | ＋ |
| *atp8* | 3,814 | 3,966 | 0 | 153 | ATT | TAA |  | ＋ |
| *atp6* | 3,960 | 4,613 | -7 | 654 | ATG | TAA |  | ＋ |
| *cox3* | 4,614 | 5,393 | 0 | 780 | ATG | TAA |  | ＋ |
| *trnG* | 5,395 | 5,459 | 1 | 65 |  |  | TCC | ＋ |
| *nad3* | 5,460 | 5,813 | 0 | 354 | ATC | TAA |  | ＋ |
| *trnA* | 5,821 | 5,882 | 7 | 62 |  |  | TGC | ＋ |
| *trnR* | 5,883 | 5,945 | 0 | 63 |  |  | TCG | ＋ |
| *trnN* | 5,945 | 6,010 | -1 | 66 |  |  | GTT | ＋ |
| *trnS1(AGN)* | 6,010 | 6,075 | -1 | 66 |  |  | GCT | ＋ |
| *trnE* | 6,079 | 6,143 | 3 | 65 |  |  | TTC | ＋ |
| *trnF* | 6,142 | 6,207 | -2 | 66 |  |  | GAA | － |
| *nad5* | 6,208 | 7,875 | 0 | 1,668 | TTG | TAA |  | － |
| *trnH* | 7,876 | 7,936 | 0 | 61 |  |  | GTG | － |
| *nad4* | 7,937 | 9,245 | 0 | 1,309 | ATG | T |  | － |
| *nad4l* | 9,239 | 9,514 | -7 | 276 | ATT | TAA |  | － |
| *trnT* | 9,517 | 9,579 | 2 | 63 |  |  | TGT | ＋ |
| *trnP* | 9,580 | 9,645 | 0 | 66 |  |  | TGG | － |
| *nad6* | 9,648 | 10,124 | 2 | 477 | ATT | TAA |  | ＋ |
| *cob* | 10,124 | 11,260 | -1 | 1,137 | ATG | TAG |  | ＋ |
| *trnS2(UCN)* | 11,259 | 11,324 | -2 | 66 |  |  | TGA | ＋ |
| *nad1* | 11,324 | 12,256 | -1 | 933 | ATA | TAA |  | － |
| *trnL1(CUN)* | 12,257 | 12,323 | 0 | 67 |  |  | TAG | － |
| *rrnL* | 12,324 | 13,527 | 0 | 1,204 |  |  |  | － |
| *trnV* | 13,528 | 13,590 | 0 | 63 |  |  | TAC | － |
| *rrnS* | 13,591 | 14,333 | 0 | 743 |  |  |  | － |
| CR | 14,334 | 15,235 | 0 | 902 |  |  |  | ＋ |

**Supplementary Table S3.** Organization of the *Scaphoideus varius* mitochondrial genome.

| Name | Start | Stop | Intergenic nucleotides | Length | Start codon | Stop codon | Anticodon | coding strand |
| --- | --- | --- | --- | --- | --- | --- | --- | --- |
| *trnI* | 1 | 67 | 0 | 67 |  |  | GAT | ＋ |
| *trnQ* | 65 | 133 | -3 | 69 |  |  | TTG | － |
| *trnM* | 141 | 206 | 7 | 66 |  |  | CAT | ＋ |
| *nad2* | 207 | 1,184 | 0 | 978 | ATA | TAA |  | ＋ |
| *trnW* | 1,186 | 1,254 | 1 | 69 |  |  | TCA | ＋ |
| *trnC* | 1,247 | 1,309 | -8 | 63 |  |  | GCA | － |
| *trnY* | 1,318 | 1,381 | 8 | 64 |  |  | GTA | － |
| *cox1* | 1,483 | 3,018 | 101 | 1,536 | ATG | TAA |  | ＋ |
| *trnL2(UUR)* | 3,022 | 3,083 | 3 | 62 |  |  | TAA | ＋ |
| *cox2* | 3,084 | 3,765 | 0 | 682 | ATA | T |  | ＋ |
| *trnK* | 3,766 | 3,835 | 0 | 70 |  |  | CTT | ＋ |
| *trnD* | 3,836 | 3,901 | 0 | 66 |  |  | GTC | ＋ |
| *atp8* | 3,902 | 4,054 | 0 | 153 | ATA | TAA |  | ＋ |
| *atp6* | 4,048 | 4,701 | -7 | 654 | ATG | TAA |  | ＋ |
| *cox3* | 4,703 | 5,482 | 1 | 780 | ATG | TAA |  | ＋ |
| *trnG* | 5,482 | 5,543 | -1 | 62 |  |  | TCC | ＋ |
| *nad3* | 5,544 | 5,897 | 0 | 354 | ATT | TAA |  | ＋ |
| *trnA* | 5,901 | 5,964 | 3 | 64 |  |  | TGC | ＋ |
| *trnR* | 5,968 | 6,032 | 3 | 65 |  |  | TCG | ＋ |
| *trnN* | 6,032 | 6,102 | -1 | 71 |  |  | GTT | ＋ |
| *trnS1(AGN)* | 6,102 | 6,168 | -1 | 67 |  |  | GCT | ＋ |
| *trnE* | 6,170 | 6,232 | 1 | 63 |  |  | TTC | ＋ |
| *trnF* | 6,233 | 6,297 | 0 | 65 |  |  | GAA | － |
| *nad5* | 6,297 | 7,964 | -1 | 1,668 | TTG | TAA |  | － |
| *trnH* | 7,965 | 8,026 | 0 | 62 |  |  | GTG | － |
| *nad4* | 8,027 | 9,335 | 0 | 1,309 | ATG | T |  | － |
| *nad4l* | 9,329 | 9,604 | -7 | 276 | ATT | TAA |  | － |
| *trnT* | 9,607 | 9,670 | 2 | 64 |  |  | TGT | ＋ |
| *trnP* | 9,671 | 9,737 | 0 | 67 |  |  | TGG | － |
| *nad6* | 9,740 | 10,219 | 2 | 480 | ATA | TAA |  | ＋ |
| *cob* | 10,219 | 11,355 | -1 | 1,137 | ATG | TAA |  | ＋ |
| *trnS2(UCN)* | 11,375 | 11,437 | 19 | 63 |  |  | TGA | ＋ |
| *nad1* | 11,439 | 12,369 | 1 | 931 | ATT | T |  | － |
| *trnL1(CUN)* | 12,370 | 12,436 | 0 | 67 |  |  | TAG | － |
| *rrnL* | 12,437 | 13,639 | 0 | 1,203 |  |  |  | － |
| *trnV* | 13,640 | 13,702 | 0 | 63 |  |  | TAC | － |
| *rrnS* | 13,703 | 14,445 | 0 | 743 |  |  |  | － |
| CR | 14,446 | 15,207 | 0 | 762 |  |  |  | ＋ |


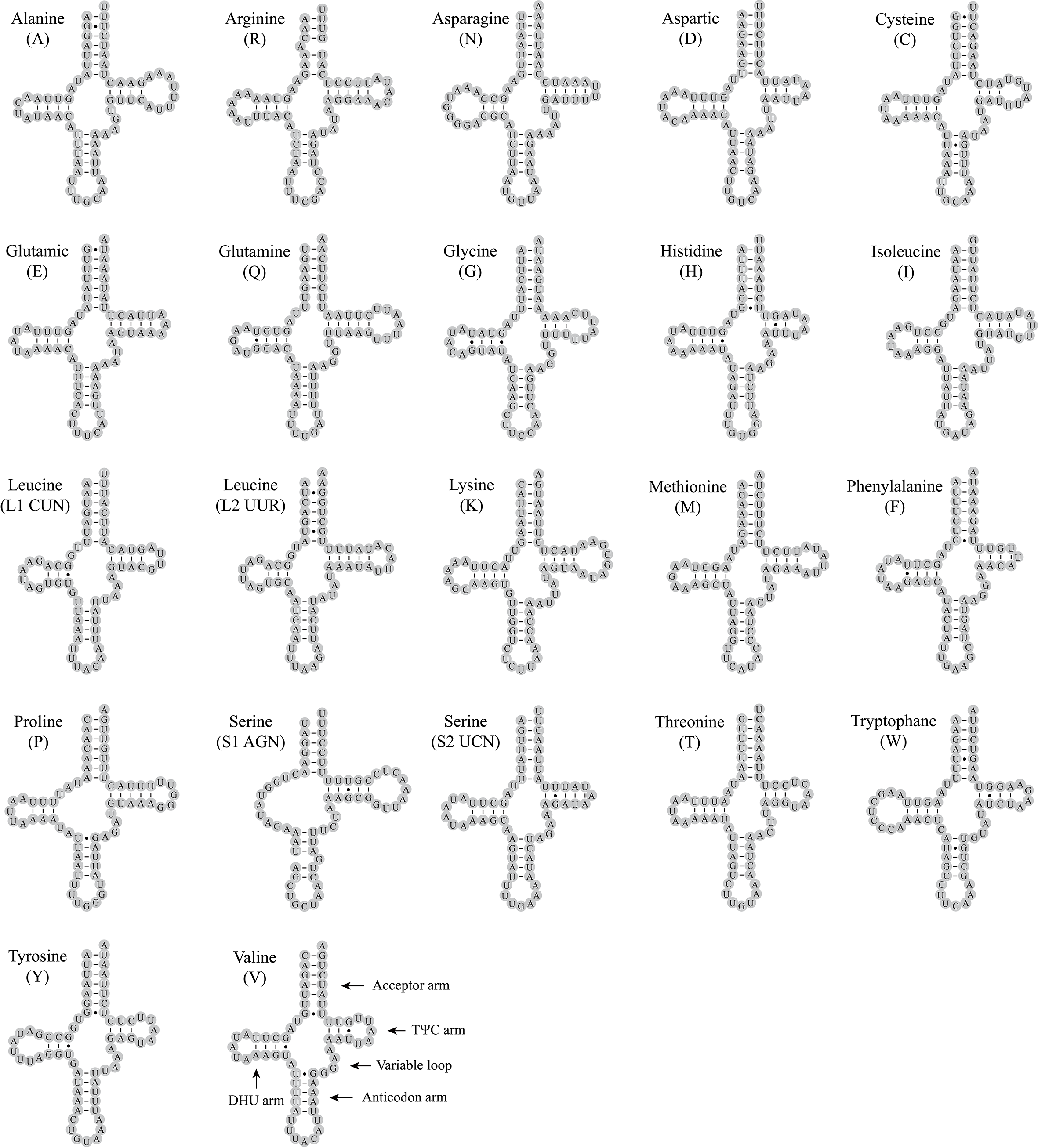


**Figure S2.** Inferred secondary structures of 22 tRNA genes in the mitochondrial genome of *Scaphoideus maai*. Watson-Crick base pairings are illustrated by lines (-), whereas GU base pairings are illustrated by dots (·).


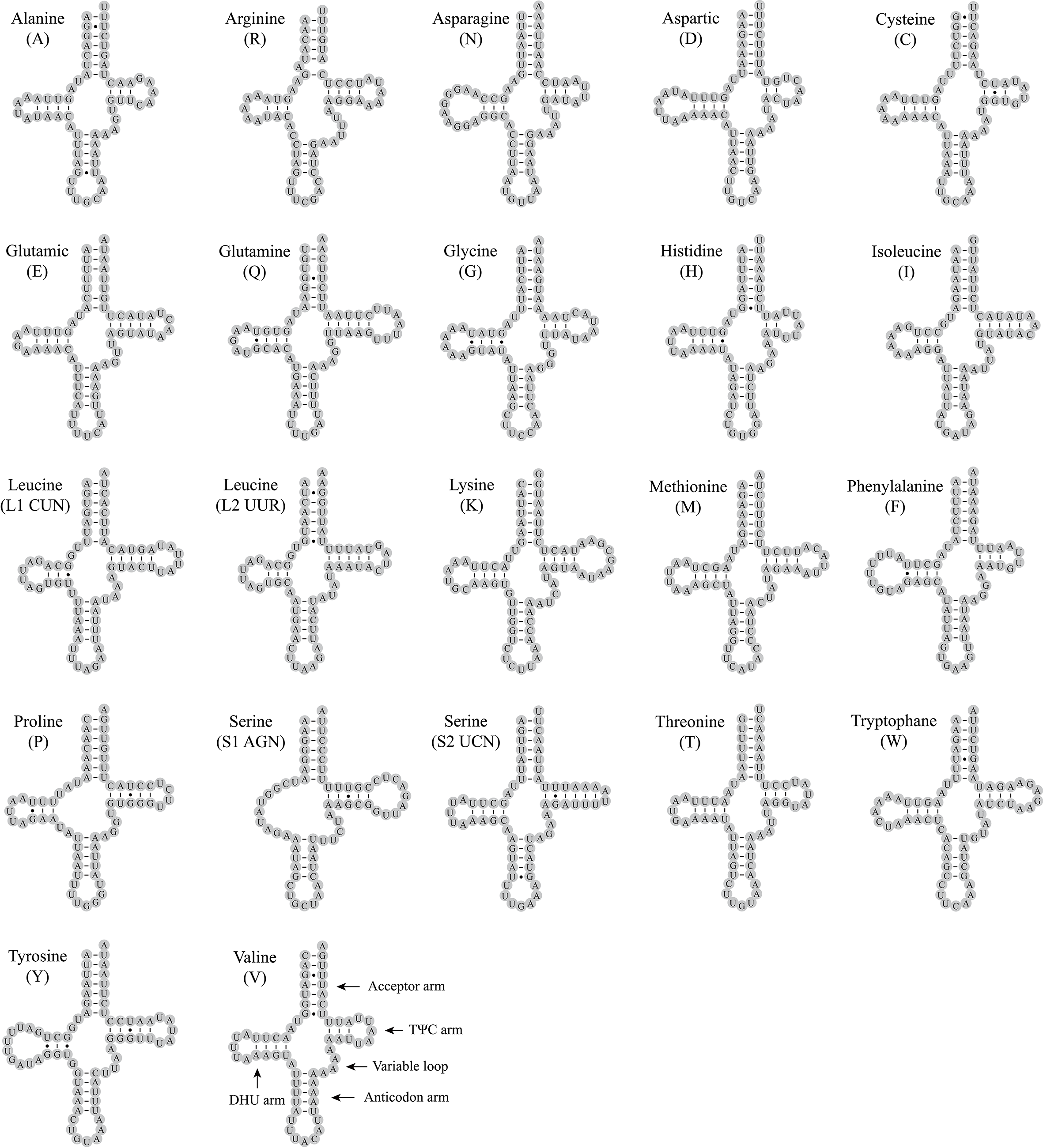


**Figure S3.** Inferred secondary structures of 22 tRNA genes in the mitochondrial genome of *Scaphoideus nigrivalveus*. Watson-Crick base pairings are illustrated by lines (-), whereas GU base pairings are illustrated by dots (·).


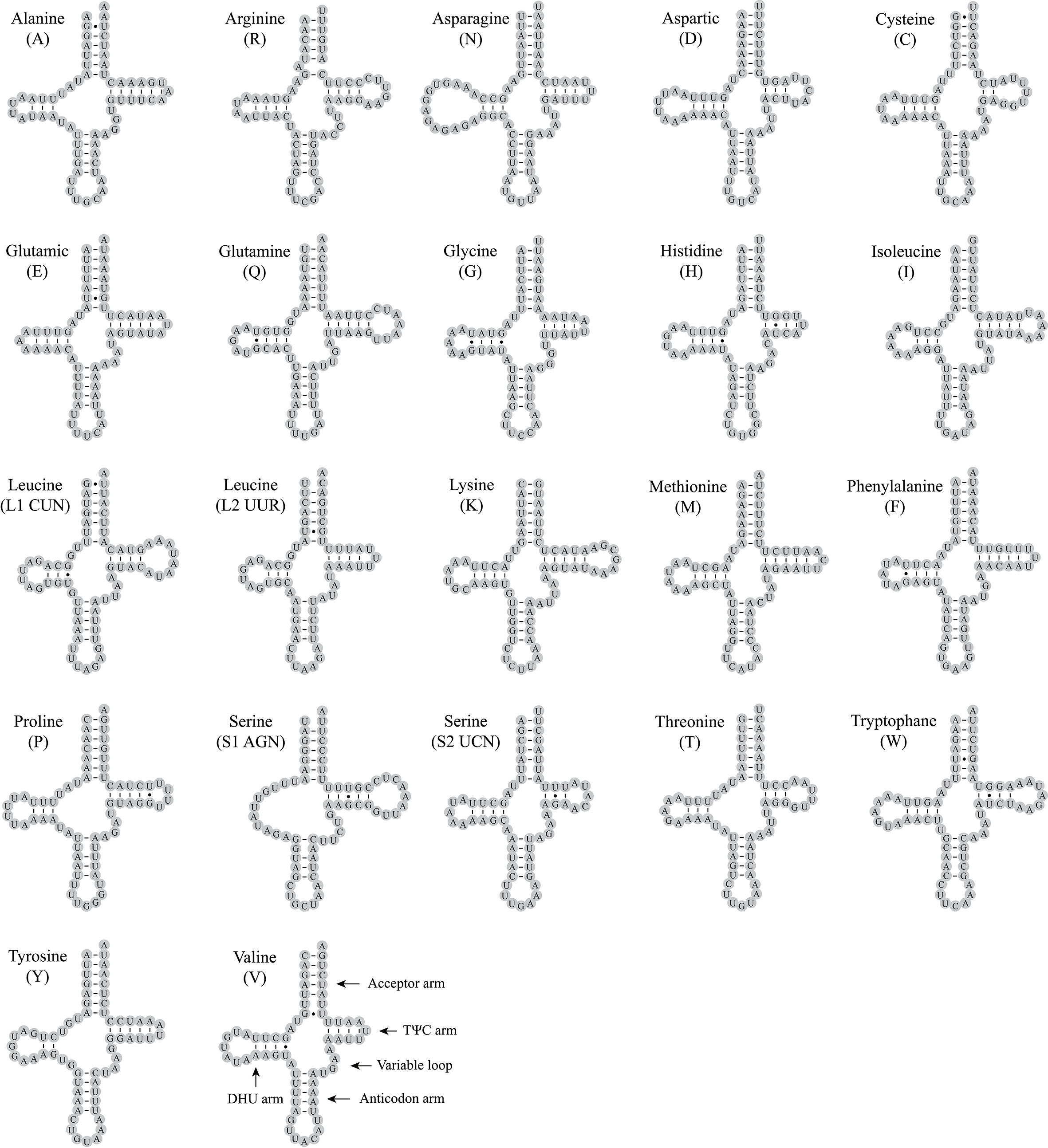


**Figure S4.** Inferred secondary structures of 22 tRNA genes in the mitochondrial genome of *Scaphoideus varius*. Watson-Crick base pairings are illustrated by lines (-), whereas GU base pairings are illustrated by dots (·).


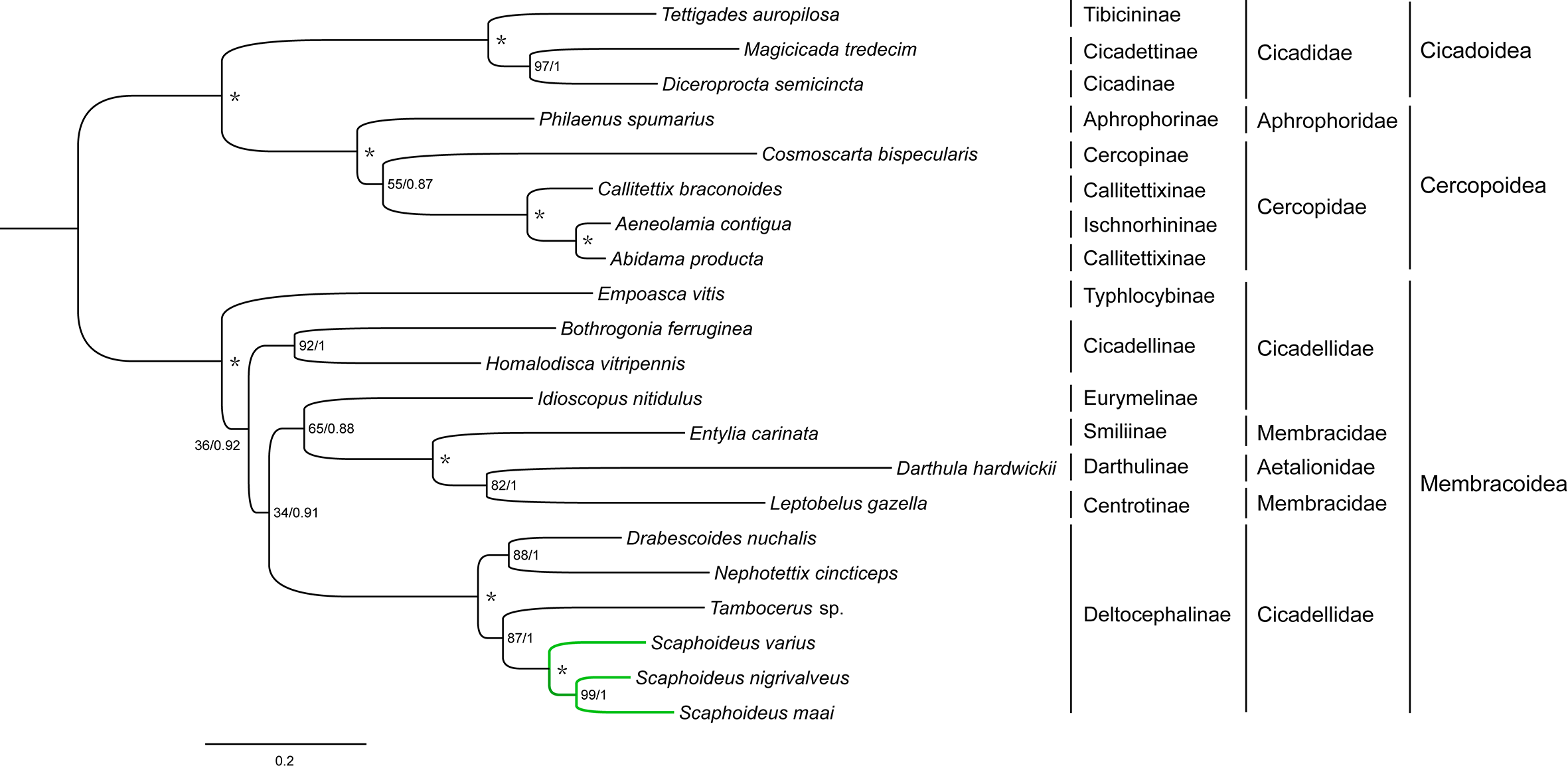


**Figure S5. Phylogenetic relationships for *Scaphoideus* based on the AA datasets inferred from RaxML and MrBayes.** Numbers on branches are Bootstrap values (BS) and Bayesian posterior probabilities (PP) respectively. An asterisk indicates BS = 100 and PP = 1.0.

**Supplementary Table S4. Collection information for *Scaphoideus* species newly sequenced in the present study.**

| **Species** | **Abbreviation** | **Locality** | **Time** |
| --- | --- | --- | --- |
| *Scaphoideus maai* | SCMA | Menghai, Yunnan, China (21°96' N, 100°45' E) | 12 July 2011 |
| *Scaphoideus nigrivalveus* | SCNI | Ankang, Shaanxi, China (32°68' N, 109°03' E) | 7 August 2014 |
| *Scaphoideus varius* | SCVA | Ankang, Shaanxi, China (32°68' N, 109°03' E) | 25 July 2015 |

**Supplementary Table S5.** Best partitioning scheme and models for different datasets selected by PartitionFinder.

| Data matrix | Subset Partitions | Model |
| --- | --- | --- |
| P123-BI | P1: (*atp6*_pos1, *cox1*_pos1, *cox2*_pos1, *cox3*_pos1) | GTR+I+G |
|  | P2: (*atp6*_pos2, *atp8*_pos2, *cox1*_pos2, *cox2*_pos2, *cox3*_pos2, *nad1*_pos2, *nad2*_pos2, *nad3*_pos2, *nad4l*_pos2, *nad4*_pos2, *nad5*_pos2, *nad6*_pos2) | GTR+I+G |
|  | P3: (*atp6*_pos3, *cox1*_pos3, *cox2*_pos3, *cox3*_pos3, *cob*_pos3, *nad2*_pos3, *nad3*_pos3, *nad6*_pos3) | GTR+I+G |
|  | P4: (*atp8*_pos1, *atp8*_pos3, *nad1*_pos1, *nad2*_pos1, *nad3*_pos1, *nad4l*_pos1, *nad4*_pos1, *nad5*_pos1, *nad6*_pos1) | GTR+I+G |
|  | P5: (*cob*_pos1, *cob*_pos2) | GTR+G |
|  | P6: (*nad1*_pos3, *nad4l*_pos3, *nad4*_pos3, *nad5*_pos3) | HKY+I+G |
| P123-ML | P1: (*atp6*_pos1, *atp8*_pos1, *cox1*_pos1, *cox2*_pos1, *cox3*_pos1) | GTR+I+G |
|  | P2: (*atp6*_pos2, *atp8*_pos2, *cox1*_pos2, *cox2*_pos2, *cox3*_pos2, *nad1*_pos2, *nad2*_pos2, *nad3*_pos2, *nad4l*_pos2, *nad4*_pos2, *nad5*_pos2, *nad6*_pos2) | GTR+I+G |
|  | P3: (*atp6*_pos3, *atp8*_pos3, *cox1*_pos3, *cox2*_pos3, *cox3*_pos3, *cob*_pos3, *nad2*_pos3, *nad3*_pos3, *nad6*_pos3) | GTR+I+G |
|  | P4: (*cob*_pos1, *cob*_pos2) | GTR+I+G |
|  | P5: (*nad1*_pos1, *nad2*_pos1, *nad3*_pos1, *nad4l*_pos1, *nad4*_pos1, *nad5*_pos1, *nad6*_pos1) | GTR+I+G |
|  | P6: (*nad1*_pos3, *nad4l*_pos3, *nad4*_pos3, *nad5*_pos3) | GTR+I+G |
| P123DEGEN-BI | P1: (*atp6*_pos1, *atp8*_pos1, *cox1*_pos1, *cox2*_pos1, *cox3*_pos1, *cob*_pos1) | GTR+G |
|  | P2: (*atp6*_pos2, *atp8*_pos2, *cox1*_pos2, *cox1*_pos3, *cox2*_pos2, *cox3*_pos2, *cob*_pos2) | GTR+G |
|  | P3: (*atp6*_pos3, *cox2*_pos3, *cox3*_pos3, *cob*_pos3, *nad1*_pos2, *nad1*_pos3, *nad2*_pos2, *nad3*_pos2, *nad4l*_pos2, *nad4l*_pos3, *nad4*_pos2, *nad4*_pos3, *nad5*_pos2, *nad5*_pos3, *nad6*_pos2) | GTR+G |
|  | P4: (*atp8*_pos3, *nad1*_pos1, *nad2*_pos1, *nad2*_pos3, *nad3*_pos1, *nad3*_pos3, *nad4l*_pos1, *nad4*_pos1, *nad5*_pos1, *nad6*_pos1, *nad6*_pos3) | GTR+G |
| P123DEGEN-ML | P1: (*atp6*_pos1, *atp8*_pos1, *atp8*_pos3, *cox1*_pos1, *cox2*_pos1, *cox3*_pos1, *cob*_pos1) | GTR+G |
|  | P2: ( *atp6*_pos2, *atp8*_pos2, *cox1*_pos2, *cox1*_pos3, *cox2*_pos2, *cox3*_pos2, *cob*_pos2) | GTR+I+G |
|  | P3: (*atp6*_pos3, *cox2*_pos3, *cox3*_pos3, *cob*_pos3, *nad1*_pos2, *nad1*_pos3, *nad2*_pos2, *nad3*_pos2, *nad3*_pos3, *nad4l*_pos2, *nad4l*_pos3, *nad4*_pos2, *nad4*_pos3, *nad5*_pos2, *nad5*_pos3, *nad6*_pos2) | GTR+G |
|  | P4: (*nad1*_pos1, *nad2*_pos1, *nad2*_pos3, *nad3*_pos1, *nad4l*_pos1, *nad4*_pos1, *nad5*_pos1, *nad6*_pos1, *nad6*_pos3) | GTR+G |
| P123R-BI | P1: (12S, 16S, *atp8*_pos1, *nad1*_pos1, *nad2*_pos1, *nad3*_pos1, *nad4l*_pos1, *nad4*_pos1, *nad5*_pos1, *nad6*_pos1) | GTR+I+G |
|  | P2: (*atp6*_pos1, *cox1*_pos1, *cox2*_pos1, *cox3*_pos1) | GTR+I+G |
|  | P3: (*atp6*_pos2, *atp8*_pos2, *cox1*_pos2, *cox2*_pos2, *cox3*_pos2, *nad1*_pos2, *nad2*_pos2, *nad3*_pos2, *nad4l*_pos2, *nad4*_pos2, *nad5*_pos2, *nad6*_pos2) | GTR+I+G |
|  | P4: (*atp6*_pos3, *atp8*_pos3, *cox1*_pos3, *cox2*_pos3, *cox3*_pos3, *cob*_pos3, *nad2*_pos3, *nad3*_pos3, *nad6*_pos3) | GTR+I+G |
|  | P5: (*cob*_pos1, *cob*_pos2) | GTR+G |
|  | P6: (*nad1*_pos3, *nad4l*_pos3, *nad4*_pos3, *nad5*_pos3) | HKY+I+G |
| P123R-ML | P1: (12S, 16S, *nad1*_pos1, *nad2*_pos1, *nad3*_pos1, *nad4l*_pos1, *nad4*_pos1, *nad5*_pos1, *nad6*_pos1) | GTR+I+G |
|  | P2: (*atp6*_pos1, *atp8*_pos1, *cox1*_pos1, *cox2*_pos1, *cox3*_pos1) | GTR+I+G |
|  | P3: (*atp6*_pos2, *atp8*_pos2, *cox1*_pos2, *cox2*_pos2, *cox3*_pos2, *nad1*_pos2, *nad2*_pos2, *nad3*_pos2, *nad4l*_pos2, *nad4*_pos2, *nad5*_pos2, *nad6*_pos2) | GTR+I+G |
|  | P4: (*atp6*_pos3, *atp8*_pos3, *cox1*_pos3, *cox2*_pos3, *cox3*_pos3, *cob*_pos3, *nad2*_pos3, *nad3*_pos3, *nad6*_pos3) | GTR+I+G |
|  | P5: (*cob*_pos1, *cob*_pos2) | GTR+I+G |
|  | P6: (*nad1*_pos3, *nad4l*_pos3, *nad4*_pos3, *nad4*, *nad5*_pos3) | GTR+I+G |
| AA-BI | P1: (*atp6*, *cox2*, *cox3*, *nad2*, *nad3*, *nad4*, *nad4l*, *nad5*, *nad6*) | MtREV+I+G+F |
|  | P2: (*atp8*, *cob*, *nad1*) | MtREV+G |
|  | P3: (*cox1*) | MtREV+I+G |
| AA-ML | P1: (*atp6*, *cox2*, *cox3*, *nad2*, *nad3*, *nad4*, *nad4l*, *nad5*, *nad6*) | MtArt+I+G+F |
|  | P2: (*atp8*, *cob*, *nad1*) | MtArt+G |
|  | P3: (*cox1*) | MtArt+I+G |
